# Oral Modified Release Multiple-Unit Particulate Systems: Compressed Pellets, Microparticles and Nanoparticles

**DOI:** 10.3390/pharmaceutics10040176

**Published:** 2018-10-04

**Authors:** Nihad Al-Hashimi, Nazish Begg, Raid G. Alany, Hany Hassanin, Amr Elshaer

**Affiliations:** 1 Drug Discovery, Delivery and Patient Care (DDDPC), School of Life Sciences, Pharmacy and Chemistry, Kingston University, Kingston upon Thames, Surrey KT1 2EE, UK; k1406374@kingston.ac.uk (N.A.-H.); ns.begg96@gmail.com (N.B.); R.Alany@kingston.ac.uk (R.G.A.); 2 School of Mechanical and Automotive Engineering, Kingston University London, Kingston upon Thames, Surrey KT1 2EE, UK; H.Hassanin@kingston.ac.uk

**Keywords:** multiparticulate, pellets, nano/microparticles, compaction, polymers

## Abstract

Oral modified-release multiparticulate dosage forms, which are also referred to as oral multiple-unit particulate systems, are becoming increasingly popular for oral drug delivery applications. The compaction of polymer-coated multiparticulates into tablets to produce a sustained-release dosage form is preferred over hard gelatin capsules. Moreover, multiparticulate tablets are a promising solution to chronic conditions, patients’ adherence, and swallowing difficulties if incorporated into orodispersible matrices. Nonetheless, the compaction of multiparticulates often damages the functional polymer coat, which results in a rapid release of the drug substance and the subsequent loss of sustained-release properties. This review brings to the forefront key formulation variables that are likely to influence the compaction of coated multiparticulates into sustained-release tablets. It focusses on the tabletting of coated drug-loaded pellets, microparticles, and nanoparticles with a designated section on each. Furthermore, it explores the various approaches that are used to evaluate the compaction behaviour of particulate systems.

## 1. Introduction

Conventional immediate-release (IR) dosage forms fail to maintain stable plasma levels of drug over a prolonged period; thus, they generally tend to have a short duration of action, which necessitates multiple daily dosing. On the other hand, multi-dose therapy is feasible for short-term conditions such as colds and flus, migraines, and neuralgia, in which treatment may last a few days. However, for long-term chronic conditions where treatment may be for several months or even years, multiple daily dosing is undesirable and inconvenient for the patient, and can result in missed doses or made-up doses [1]. In the United States (US), it is estimated that billions of dollars are spent every year because of the consequences of patient noncompliance. Hospitalisation costs as a result of patients being noncompliant with their medication are as high as $13.35 billion a year. However, it is not just a financial burden; patients who choose to ignore medical advice given to them or forget to take their medication (which is more common with geriatric patients) risk worsening their condition or in some cases reducing their chances of survival. It is estimated that around 125,000 deaths are caused every year as a direct result of nonadherence to medication. Martin et al. conducted a separate study; patients suffering from hypertension were placed on a thrice daily dosing regimen and a once-daily regimen. Patient adherence to the thrice-daily schedule was 59% whereas for the once-daily regimen, around 84% of patients complied with the medication schedule [2].

Oral modified-release dosage forms are becoming increasingly more popular and effective alternatives to IR solid oral dosage forms. The objective of these dosage forms, in the simplest of terms, is to achieve a slow release of the drug over an extended period of time, and therefore is also referred to as an extended-release (ER) or sustained-release (SR) system [3].

Modified-release dosage formulations are classified into two distinct categories. They can be administered orally in single-unit dosage forms (SUDF) or multiple-unit dosage forms (MUDF), which are also referred to as multiparticulate dosage forms. Single-unit formulations consist of the drug substance within a single tablet or capsule, whereas multiple-unit dosage forms consist of multiparticulates of several small discrete particulates, such as pellets, microparticles, or nanoparticles combined into a single dosage unit [4]. To act as an effective sustained-release dosage form, multiparticulates are coated with a polymer, and these coated multiparticulates are then either compressed into tablets or filled into hard gelatin capsules as the final dosage form [5].

There is now growing interest in the compaction of coated multiparticulates into sustained-release tablets as opposed to hard gelatin capsules. Tablets are much easier and cheaper to produce with a higher production speed. There has been an emphasis on the development of tabletting technology as one of the most common solid oral dosage forms. Tablets are small, mechanically strong, and can be divided into subunits, which is impossible to achieve with hard gelatin capsules. The compaction of coated multiparticulates into tablets reduces the risk of product tampering, which is a major concern with capsules. Also, geriatric patients have difficulty in swallowing hard gelatin capsules, which is most likely due to the large capsule size, so this has an impact on patient compliance to medication [6]. Adding multiple-unit dosage forms into an orodispersible matrix can offer far more advantages as a sustained release oral formulation, especially to dysphagia patients. Upon the oral administration of a sustained release tablet consisting of coated multiparticulates, the rapid disintegration of the tablet matrix is expected, and this should release the individually coated particulates, which may be pellets, microcapsules, or nanocapsules. The small size of such multiparticulates would enable better spreading and distribution over a large surface area along the gastrointestinal tract. This prevents high local drug concentrations and thus a reduction in potential local irritation (especially with weak acids). In multiple-unit dosage forms, the drug substance or active pharmaceutical ingredient (API) is present in each discrete coated particulate, and so therefore, the total drug is divided into these individual subunits. This system reduces the risk of adverse side effects and the toxicity that is associated with ‘dose dumping’ [7,8,9]. Nonetheless, the compression of the particulate systems during tablet manufacturing might be challenging [10]. The aim of the current review is to critically appraise the previous studies that looked at the compression of particulate systems into multiple-unit tablets and provide an insight into future developments in this field. Three different types of polymer-coated particulates—pellets, microparticles, and nanoparticles—will be surveyed. Significant formulation parameters are discussed, including the type of coating polymer used (e.g., cellulosic or acrylic), the quantity of polymer coating used, the effect of plasticisers and excipients in addition to other process variables such as the effect of compression force on protecting the integrity of the polymer coat. The review also briefly summarised the various compaction theories that can be used to understand the compaction behaviour of particulate systems.

## 2. Compaction Process

During the compaction process, powder is compacted, and its status changed into tablets of a required porosity. The compaction is affected by the physical properties of the powder (such as particle size and morphology), powder mixture, and compaction forces, in addition to the effect of the process condition [11].

By applying pressure over powder granules, the powder volume and the amount of air between the powder particles are decreased through an endothermic process [11]. During the process, a number of bonds could be formed between the powder particles; many factors affect the type of the bond formed, such as the amount of pressure applied and the powder properties. When the particles of the powder become closer to each other due to compaction, interparticulate bonds form between the individual particles, which is accompanied by heat release [11].

On the other hand, the compaction of pellets and granules is a two-stage process. In the first stage, the pellets are rearranged and deformed, whilst the second stage involves crushing the pellets. The latter is affected by the pellets’ porosity and deformability [12]. Besides, the granules’ preparation process affects the compaction behavior of the prepared pellets. Granules prepared by dry granulation processes always have deteriorated compressibility and compaction properties because of work hardening [13].

### Challenges Associated with Compression of Coated Multiparticulates

This section focuses on the the compaction of reservoir-type multiparticulate drug delivery systems (coated multiparticulates) into sustained release tablets and the subsequent drug release behaviour of these dosage forms, which ideally should not be affected by the compaction process. The polymeric coating surrounding each drug-loaded particulate must retain its integrity, withstand the compaction force, and remain intact and free of cracks.

Damage to the coating can significantly alter the release/dissolution behaviour of the drug and cause a rapid release of the drug substance, therefore resulting in the loss of the controlled release properties.

## 3. Coated Pellets (Reservoir-Type Multiparticulate Drug Delivery Systems)

**Figure 1:**
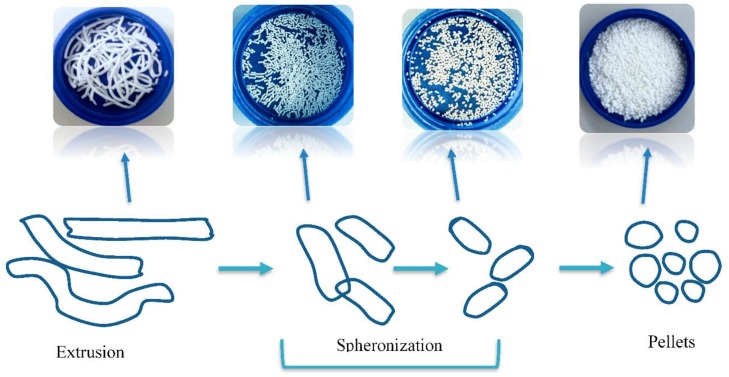
Diagram showing how drug-loaded pellets are prepared using an extrusion spheronization technique.

Tabletted pellets comprise several individual coated spherical granules, which are referred to as pellets, with a size range typically between 0.5–1.5 mm [14]. Each individual pellet contains the drug substance where the total drug dose is divided. There are two main methods by which coated pellets (reservoir systems) are prepared for compression into tablets. Extrusion–spheronization, as shown in Figure 1, is the process by which the dry powder mixture (consisting of the active pharmaceutical ingredient (API) and excipients) is agglomerated with the aid of a binding fluid. This mass is then processed in an extruder whereby extrudates are produced. Finally, the spheronizing stage converts these extrudates into spherical granules, i.e., pellets. These spherical drug-loaded pellets are then coated with polymer, followed by compression into a multiple-unit pellet system tablet [14,15].

**Figure 2:**
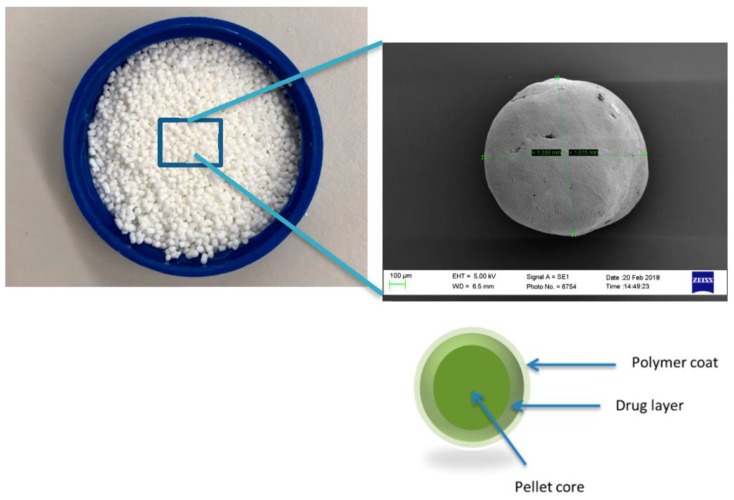
Schematic representation of tablet comprising of coated pellets.

Alternatively, nonpareil seeds (NPS) are used. These are inert cores, which are primarily composed of lactose and starch onto which the drug is layered. The modified-release polymeric coating is then layered on top of the drug coating, as shown in Figure 2, and subsequently, these pellets are compressed into sustained release tablets [15,16].

### 3.1. Compression of Coated Pellets into Tablets

#### 3.1.1. The Type of Coating Layer

The choice of polymeric coating for drug pellets is essential, as these coated pellets undergo compression to form tablets. Therefore, it is important that the polymeric coating can withstand the compression force by having the right combination of plasticity, elasticity, and thickness without rupturing. Damage to the coating polymer can compromise the sustained release properties, thus resulting in the fast release of drug (dose dumping).

The polymer coatings can be formulated as either aqueous polymeric dispersions or organic solutions. Aqueous dispersions are preferred, as they avoid the flammability and toxicity hazards associated with organic solutions, and they are also a cheaper alternative [17].

##### Cellulosic Polymers

The most common cellulose-based polymer used for sustained release dosage forms is ethyl cellulose (EC) [18]. However, EC is known to be a brittle polymer with inappropriate mechanical properties. It has a low puncture strength and low elongation (<5%) [19]. Other polymers (semisynthetic and synthetic) with different mechanical properties have been used (Table 1).

Hosseini et al. repared EC-coated propranolol hydrochloride pellets and incorporated a cushion layer to act as a surrogate to absorb the energy of compaction to help protect the EC polymeric coating, so that it remains intact during compression. Standard tabletting excipients, such as microcrystalline cellulose (MCC), lactose, or sorbitol, were layered onto the ethyl cellulose-coated pellets to form the cushion layer. Upon compression, the cushion-layered pellets ruptured, and the integrity of the EC coating was compromised, leading to an uncontrolled drug release. However, by incorporating a glidant, such as magnesium stearate, to the cushion layer, the mechanical strength of the pellet was improved, and the rupturing tendency of the polymer coat was reduced during compression [24].

Sawicki and Mazgalski developed a tabletting method to overcome the brittleness of the EC film that is used to coat the pellet core. They used hot tabletting to compress the EC-coated pellets into tablets, which use a lower compression force in comparison to the normal tabletting process. Tramadol hydrochloride pellets were coated with Aquacoat^®^ ECD. These coated pellets were then compressed into tablets using the hot tabletting method where a compression force of 1 kN was used. Polyethylene glycol3000 was a vital component that was incorporated during the tabletting process to provide additional protection to the tablets and ensure that the EC polymer coat remains intact during compression. The results revealed a successful sustained release of tramadol hydrochloride from the palletised tablets, which was attributed to the intact polymer coat. Therefore, hot tabletting can be considered as an alternative to the traditional tabletting procedures to manufacture tabletted pellets using EC as the sustained release film [25,26].

Microcrystalline cellulose (MCC) is considered the gold standard for pellets’ preparation by extrusion spheronization, as it provides appropriate plasticity to the wet mass [17]. Since the 1990s, MCC has been widely used in preparing multi-unit tablets. MCC also offers superior compaction and disintegration properties [27]. On the other hand, MCC was found to prolong the drug release, especially for poorly water-soluble drugs, as the polymer does not disintegrate quickly. MCC was found to be incompatible with some drugs (and adsorb some drugs to the surface of its fibers [14,28]. In the study of Abbaspour et al., at least 10% MCC was used to aid the pellets’ formulations process to obtain Eudragit RS/RL pellets with 60% drug loading [29].

Johansson and Alderborn evaluated the degree of deformation of MCC pellets during compaction. It was concluded that the MCC pellets did not fragment under compression, and the pellets’ porosity was the main factor affecting the structural deformation of the pellets [30]. Another study conducted by Nicklasson et al. compared the compaction behavior of MCC pellets and pellets made of 20% MCC and 80% dicalcium phosphate dehydrate (DCP). DCP/MCC pellets were rigid and less prone to densification. These pellets were extensively deformed from their surfaces and formed tablets with weak mechanical properties. On the other hand, MCC pellets formed tablets with high tensile strength [31]. Kallai et al. investigated the effect of pellets’ core materials on the mechanical properties and release kinetics of their tabletted pellets. MCC, isomalt, or sugar were used as inert cores. It was noted that the drug release profile was affected by the core material used. MCC-tabletted pellets managed to sustain the release of diclofenac sodium, which was attributed to the ability of MCC to swell in water. On the other hand, sugar and isomalt-based pellets showed faster release profiles, as the core was water-soluble and dissolved quickly. Besides, the tensile strength of MCC pellets was higher than that of sugar and isomalt pellets [32].

Hydroxy propyl methyl cellulose (HPMC) is a water-soluble polymer that was used as a binder in coated pellets. HPMC was used as a binder to load omeprazole into pellets with a sugar core or MCC core. HPMC is also known as a pellets-hardening agent, as it is able to generate durable pellets [33,34]. In a study conducted by Nguyen et al., HPMC was used as a sealing agent, and pellets that hardened with HPMC showed superior physical strength. Increasing HPMC concentrations increased the ability of the prepared pellets to resist compaction pressure. Besides, HPMC was found to delay glipizide release by creating a lag time. HPMC was also reported as a pore-forming agent in pellets manufacturing. The polymer was used at 7.5% *w/w* in studies to form pores through which water penetration was achieved to enable the drug release [35].

##### Acrylic Polymers

Acrylic polymers are generally preferred over cellulosic polymers due to their elasticity (Table 1); hence, they are less likely to rupture under compression when used to coat pellets. The elasticity of these polymers is reflected in their elongation values. Eudragit NE 30 D is an aqueous copolymer dispersion of ethyl acrylate and methyl methacrylate in a 2:1 ratio with an elongation value ≥365%. This indicates that the polymer is very flexible (has high elasticity) and has the ability to withstand deformation forces during compression [22]. Also, due to the high elasticity, there is no need to use plasticisers to increase the flexibility of the film. Plasticisers are also not required to decrease the minimum film forming temperature (MFT) of Eudragit NE 30 D, as it has a low MFT value of ~5 °C. The lack of strong interchain hydrogen bonds within the molecular structure of Eudragit NE 30 D (Table 1) contributes to the flexibility of the polymer. Eudragit^®^ RS and RL30 D, which are aqueous copolymer dispersions of acrylic acid and methacrylic acid esters, have elongation values of <50%, without the addition of a plasticiser or even with small amounts of plasticiser (10% *w/w*). However, with the addition of more plasticiser (~20% *w/w*), the elongation at break increased to give values within the range of 80–300%. Polymer films with elongation values within this range could withstand the compressive forces experienced during the tabletting process [4]. 

Akhter and Kibria studied the drug release rate-retarding properties of three acrylic polymers used to coat ambroxol hydrochloride sustained-release pellets, and the effect of these polymers on the release kinetics of the drug from the coated pellets. The three acrylic polymers investigated were Eudragit RL 30 D, Eudragit RS 30 D, and Eudragit NE 30 D, and two different media were used to perform the in vitro dissolution study: acid media (pH 1.2) and buffer media (pH 6.8). In the 0.1N-HCl (acid media), ~35% of the drug was released from Eudragit RL 30 D-coated pellets, ~13.8%(*w/w*) from Eudragit RS 30 D-coated pellets, and ~2.4%(*w/w*) from Eudragit NE 30 D-coated pellets at the first hour. The initial high drug release from the Eudragit RL 30 D-coated pellets may be due to the high-water permeability of the film causing the drug to dissolve. In contrast, the low percentage of drug release within the first hour for both Eudragit RS 30 D and Eudragit NE 30 D was attributed to the low water permeability properties of both polymers. The presence of carboxylic groups in the film as well as the swelling of the pellets within the dissolution media may have also contributed to Eudragit NE 30 D-coated pellets release-retarding properties. In the buffer media at the first hour, ~54% of the drug was released from the Eudragit RL 30 D-coated pellets, with only 7.3% and 1.1% released from the Eudragit RS 30 D and Eudragit NE 30 D-coated pellets, respectively. Overall, Eudragit NE 30 D showed the best drug release-retarding effect in comparison with the other two polymers, while Eudragit RL 30 D showed the minimum drug-release retarding effect [36]. Lunio et al. prepared tabletted pellets by compacting verapamil hydrochloride (VH) floating pellets coated with Eudragit NE 40 D using a single-stroke and rotary tablet press. Eudragit NE 40 D—just like Eudragit NE 30 D—is a neutral copolymer ester consisting of ethyl acrylate and methyl methacrylate, which is highly flexible and does not require the addition of plasticisers. When the coated pellets were compressed at a force of 6 kN, there was the sustained release of VH from the resultant tablets for approximately 6 h. This could be attributed to the high elasticity of the Eudragit NE coating, which is able to withstand the compressive force of the tabletting process. Although there was some prolonged release of the drug, this was still insufficient, and in vitro dissolution studies proved that there was a fast release of VH from the tablets, which was further explained by the scanning electron micrographs (SEMs). SEMs revealed a clear damage to the tablets where the film coating was ruptured, leading to an increase in VH release from the pellets. This was mainly attributed to the punch pressure of the single-stroke tablet press, which only has a one-stage main pressure, unlike the rotary press, which consists of a two-stage compression cycle (pre and main compression phases). When the coated pellets were compressed using a rotary tablet press with compression forces of 6 kN, 12 kN, and 18 kN, there was correlation between the mean dissolution time (MDT) and compression force. An increase in the punch compression force resulted in a longer MDT and thus a decrease in the release rate of VH. Also, there was an increase in both tablet hardness and ejection force from the die, with an increase in the compression force. The SEM images that were obtained showed a lack of damage to the Eudragit NE 40 D coat as well as to the pellet core when the coated pellets were compressed at 18 kN. Therefore, the Eudragit NE 40 D polymer coat remained intact upon compression, and the pellets deformed rather than fragmented, resulting in a sustained release of VH [18]. The study attributed this to the unsymmetry of the applied force on the pellets using the single-stroke tablet press. On the other hand, the authors argued that the rotary tablet press uses a low turret rotation speed and a longer compression time, resulting in a uniform distribution of force on the pellets’ surface, hence minimal damage.

##### Polyvinyl Acetate

Kollicoat^®^ SR 30 D is an aqueous polymer dispersion of polyvinyl acetate. It consists of polyvinyl acetate (27.0% *w/w*), povidone K 30 (2.7% *w/w*), sodium lauryl sulfate (0.3% *w/w*), and water (70.0% *w/w*). The application of Kollicoat^®^ SR 30 D onto pellets results in pH-independent sustained-release formulations. The physicochemical properties of Kollicoat^®^ SR 30 D render it a favoured choice as a coating polymer. It has a low viscosity, which allows it to be atomised into fine droplets. It is non-sticky, and therefore can be applied easily, and has a minimum film-forming temperature (MFT) of 18 °C that can be further lowered by addition of plasticisers, which also improves the flexibility of the film [37].

Dashevsky A. et al. prepared propranolol HCl pellets coated with Kollicoat^®^ SR 30 D and assessed the compressibility of these coated pellets. Ideally, Kollicoat^®^ SR 30 D coated pellets should compress into tablets with the polymer coat remaining unruptured, allowing for a slow and sustained release of the drug. The dissolution results of Dashevsky et al. showed a slight prolonged release of drug from the tabletted pellets; however, the drug release percentage was high when compared to the drug release from uncompressed pellets, which is likely to have been caused by rupturing of the Kollicoat^®^ SR 30 D polymer coating during compression. When a small amount of triethyl citrate 10% (*w/w*) was incorporated within the polymer film coating as a plasticiser, the flexibility of the polymer coat improved significantly, and this was reflected in the drug release profile. The percent drug release was less than that of the uncompressed pellets, and there was a more prolonged release of drug, indicating that Kollicoat^®^ SR 30 D with a 10% Triethyl citrate (TEC) coat remained intact during compaction [23,38]. 

#### 3.1.2. Characteristics of the Coating Layer

Generally, a thicker polymer coating increases the mechanical strength of the coated pellets, helping to protect the film integrity during compaction into tablets. A thicker coating is also believed to help improve the plastic and elastic deformation characteristics of pellets [22]. Therefore, this prevents rupturing of the polymer coat upon compaction, and allows for a slow and sustained release of the active pharmaceutical ingredient once the tablet has disintegrated to release the individual drug-loaded pellets.

Sawicki and Lunio developed floating pellets containing VH (40 mg) along with other excipients. The pellets were coated with Kollicoat^®^ SR 30 D along with the addition of propylene glycol as a plasticiser to enhance flexibility. Two polymer films of different thicknesses were used: film A (35 μm in thickness) and film B (50 μm in thickness), and the compressibility of these polymeric films was assessed. The study showed that pellets with a film thickness of 35 μm were unable to withstand the pressure force exerted during tabletting, and were thus deformed. The polymer coating may have also ruptured as a result of deformation, which caused a fast drug release from the tabletted pellets, and therefore, the sustained-release properties of the polymer coat diminished. In contrast, coated pellets with a thickness of 50 μm resulted in a slower release of VH from the tabletted pellets [39]. This meant that the thicker polymeric coating was able to better withstand the compression force of the tabletting process.

Further research was performed by Sultana et al., in which sustained-release pellets were incorporated with salbutamol sulphate as the model drug. Two different aqueous polymeric dispersions were used to coat the pellets: Eudragit RS^®^ 30 D and Kollicoat SR^®^ 30 D. The drug-containing cores were coated with different thicknesses/polymer loads (5%, 10%, 15%, 20% and 25% *w*/*w*). The study concluded that the rate of drug release was dependent upon the thickness of the coating polymer. Accordingly, a higher percentage *w*/*w* of polymer led to a slower rate of drug release. Increasing the thickness of the polymer film also decreased the porosity of the film coating, therefore retarding drug release [40]. It can be noticed from the obtained results that the properties of the coating layer have impacts that should be considered during pellet preparation.

#### 3.1.3. Role of Plasticisers

Plasticisers are additional components incorporated within the polymer coating to enhance film-forming properties, as most polymers are brittle at room temperature, so upon compaction, they are likely to damage or rupture, resulting in the faster release of the drug from the tabletted s; EC-based coatings are a prime example of such behaviour. Plasticisers are employed to increase the elasticity of the polymer film and thus film elongation, reduce moisture permeability, and decrease the MFT. Common plasticisers employed are triethyl citrate propylene glycol (PPG) [4,41].Abbaspour et al. developed ibuprofen pellets coated with Eudragit RS 30 D and Eudragit RL 30 D aqueous polymeric dispersions in a 4:1 ratio. Varying proportions of triethyl citrate were also incorporated as a plasticiser; the amounts included were 10%, 20%, and 30% (*w*/*w*). The plasticiser amount was varied to determine the optimum plasticiser amount that is required to increase the elasticity, and thus the elongation of the polymer film, so that they do not rupture upon compression. It was concluded that 20% TEC was the optimum plasticiser concentration to be incorporated within the coating formulation. In general, as the percentage of the plasticiser incorporated within the film coating was increased, so too was the elongation of the film coating. However, the stress at break decreased. So, although 30% TEC had the highest percentage elongation, it had the lowest stress at break. Meanwhile, 10% TEC had the highest stress at break, but the lowest percentage elongation. Therefore, 20% TEC was chosen to be incorporated within the coating formulation, as it had the right balance of elongation and stress at break. Upon compaction of the Eudragit RS 30 D/RL 30 D (including 20% TEC) coated ibuprofen pellets, it was concluded that the they had sufficient mechanical strength to withstand the compaction force of the tabletting press, and so drug release was successfully sustained [29].

On the other hand, Osei-Yeboah et al. produced a plasticised top coating polymer layer to effectively protect the functional polymer coating of drug-loaded pellets from any damage caused by compaction into multiple-unit pellet system (MUPS) tablets. Pyridoxine-loaded pellets were coated with EC aqueous dispersion, and these coated pellets were then further top-coated with polyvinylpyrrolidone (PVP) K30 as an additional plasticised protective layer. These top-coated pellets were then subsequently compressed into tablets. Varying amounts of PVP K30 (10%, 15%, and 20% *w*/*w*) were applied onto the functional polymer coat to assess whether a thicker plasticised layer would further protect the EC coat when compressed, and hence allow for a slow pyridoxine release over a prolonged period of time. Drug release from compressed PVP K30 top-coated pellets was compared with compressed pellets without the additional PVP K30 layer to assess the effectiveness of the protective coating. From in vitro dissolution studies, it was concluded that the PVP K30 plasticised top coating successfully sustained the release of pyridoxine from the coated pellets with minimal release over the two-h period investigated, and thus maintained the functional EC coating intact when compressed. This was not the case for coated pellets without the additional PVP K30 layer, where a rapid release of pyridoxine was reported within the 2 h as a result of the EC coat rupturing upon compaction [42].

#### 3.1.4. Effect of Pellets’ Porosity on Compression Behaviour

Both intergranular (air voids between pellets) and intragranular (air voids within pellets) porosity impact the characteristics and performance of pharmaceutical pellets. Porosity has profound effects on the compression mechanism, and therefore the polymer coat integrity and tensile strength of the resulting tablet formed after compression. Generally, highly porous pellets undergo an increased degree of deformation (change in pellet shape) during compression, which leads to slightly flattened pellets. However, they are not fragmented, and therefore, the polymer coat does not rupture, and the resulting tablets have high tensile strength due to the presence of strong intergranular bonds, and drug release will be unaffected. This is also the case with the high intragranular porosity of pellets [43].

Tunón et al. investigated the compression behaviour and the resulting drug release rate of three batches of pellets with varying intragranular porosities (low, intermediate, and high porosity). These varying porosities were achieved by using different proportions of ethanol and water in the granulation liquid, and the pellets were coated with a thin layer of EC. The study concluded that the more porous pellets were much less affected by the compaction force of the tabletting process, and therefore maintained the integrity of the polymer coat, and a sustained drug release was not impaired. Pellets with high porosity gave rise to increased deformation and densification upon compression, and so the sustained release of the drug was unaffected. Whereas pellets with low porosity were only slightly deformed and densified, and so drug release was relatively high [44]. These results come in accordance with Johansson and Alderborn’s study that evaluated the MC granules of two types—irregular and spherical shapes—with different original intragranular porosities (low, intermediate, and high) [30].

#### 3.1.5. Effect of Pellet Size on Compression Behaviour

The pellet size can influence their compaction mechanisms/properties, and therefore the polymer coat integrity and drug release from the tabletted pellets.

Dashevsky et al. investigated the effect of pellet size on propranolol HCl release from pellets coated with aqueous polymer dispersion, Kollicoat^®^ SR 30 D. They found that smaller pellets had a larger surface area than the larger pellets at the same weight, and therefore had a thinner coating. So, upon the compression of these smaller pellets, fragmentation occurred, and the polymer coat ruptured. This would have a direct impact on drug release, as the polymer coat integrity is comprised, drug release tends to be faster, and so the sustained drug release properties of these tabletted pellets are no longer applicable. It was concluded that propranolol HCl release from the coated pellets was decreased with the increase in pellet size. The larger pellets did withstand the compaction force of the tabletting process, and so, the polymer coat remained unruptured, and the drug release rate slowed down [23,38]. On the other hand, other studies conducted by Johansson et al. and Wu et al. suggested otherwise. Where Johansson et al. looked at two sets of pellets with different size fractions/different diameters; the first pellet fraction had a diameter of between 425–500 μm (small pellets) and the second fraction had a diameter of between 1250–1400 μm (large pellets). They found that pellet size before compaction influenced the degree of deformation upon compaction. The larger pellets increased the degree of deformation, and this was further increased by increasing the compression pressure. Tablet tensile strength was increased when the applied pressure of compaction was also increased, and this was more pronounced with the larger pellets [45]). Similarly, Wu et al. found that larger pellets were more fragile than smaller ones. By measuring the bulk density of smaller and larger pellets, it was found that the greater the bulk density, the higher the compressibility [46].

#### 3.1.6. Effect of Pellets’ Composition on their Compression Behaviour

Generally, when stress is applied to pellets or granules during the process of compression, two types of deformation exists: elastic deformation (reversible) and plastic deformation (non-reversible). Elastic deformation refers to a reversible or temporary shape change where particles regain their shape once the applied stress is removed. Plastic deformation refers to an irreversible or permanent shape change once the applied stress is removed. For elastic deformation to occur, low stress must be applied, and this is not feasible for tablet manufacturing, as the stress applied during the compression process is much greater than that required for elastic deformation. If low stress was to be applied during the tablets’ manufacture, the resulting tablets would be brittle. Therefore, plastic deformation is necessary to produce a mechanically durable compressed tabletted pellets as a successful sustained-release dosage form, and the choice of excipients/components incorporated within the pellet core is vital. These excipients must be able to undergo plastic deformation themselves to aid in producing a successful tabletted pellet [47].

Excipients incorporated within the pellet core along with the drug substance are mostly diluents (also referred to as fillers) and binders. Common excipients include MCC, PVP, hydroxypropyl methyl cellulose (HPMC), lactose, and starch [48]. A recent study conducted by Chin, Chan, and Heng suggested that the particle size of the lactose as a cushioning layer influenced the integrity of the compressed pellets. Pellets compressed with coarse lactose particles had deeper and larger indentation, whilst micronized lactose managed to protect the pellets from damage [49].

MCC is the most common excipient incorporated within pellet formulations, mainly because it can undergo plastic deformation during compression, the importance of which has already been discussed. It also has good binding properties, giving rise to pellets that are both strong and cohesive, therefore making them more suitable for compaction into tablets. Due to having the right combination of plasticity, cohesiveness, and rheology, MCC is the gold standard component for the manufacture of pellets [22,50]. 

Also, MCC showed good compaction properties when used to make metronidazole tablets. It was found that MCC and HPMC have good compaction properties and can be used to improve the compaction of weak compactable powders, and produce modified release tablets [11].

Although MCC already has a high degree of plasticity, incorporating it with other excipients within the pellet core can further increase the compressibility of pellets, and therefore maintain integrity and deform without fragmenting during tabletting [51].

Whilst MCC is regarded as the gold standard excipient in terms of pellet formulation, there are some exceptions. Such exceptions include drugs being adsorbed onto the surface of MCC fibers, drugs being chemically incompatible with MCC, and MCC powders from different suppliers with different properties [50]. Krueger et al. evaluated the use of MCC I polymorph (MCC II) as a new pelletisation aid in extrusion/spheronisation. The study evaluated the effect of process variables such as spheroniser load, spheronisation speed, and time on the properties of MCC II pellets in comparison with MCC I. Both spheronisation time and speed were found to have a significant impact on the characteristics of the formed MCC II pellets. The aspect ratio and porosity were found to decrease during the spheronisation process, whilst the pellet weight and diameter increased during the process. On the contrary, MCC I pellets behaved differently, as their weight remained constant, and the pellets equivalent diameter decreased [52]. Besides, other excipients were evaluated as a substitute for MCC in the extrusion spheronisation, such as for instance chitosan, pectinic acid, polyethylene oxide, HPMC, and k-carrageenan [53].

Bornhöft et al. investigated the use of carrageenan as a potential substitute for MCC as a pelletisation aid. Three different types of carrageenan were investigated: ι-, κ-, and λ-carrageenan, all of which are available commercially. It was concluded that both the ι- and λ-carrageenans were unsuitable excipients for the preparation of pellets, as they resulted in unrounded pellets that were of low mechanical strength, too brittle, and of insufficient plasticity, and so would easily fragment upon compaction, thus rupturing any polymer coating and resulting in a burst release. However, к-carrageenan pellets were of high mechanical strength, and had a good degree of plasticity, hence, they are considered to be a promising alternative to MCC in the manufacturing of pellets [54].

Another study conducted by Thommes and Kleinebudde investigated the suitability of κ-carrageenan as a pelletisation aid when used in combination with different fillers. Four different fillers were used—lactose, mannitol, starch, and dicalcium phosphate—and 20%, 40%, and 60% fractions of each filler were added into the powder mixture. The κ-carrageenan pellets that were produced were spherical in shape, had a narrow pellet size distribution, and were of sufficient mechanical strength to be coated with polymer and compressed into tablets. The incorporation of stearic acid as a cushioning agent was investigated [55].

Pellets containing stearic acid were protected from compression-induced damage without manipulating the disintegration profile of these formulations. Yang et al. utilized talc in the coating dispersion of Eudragit NE 30 D pellets in order to maintain their integrity and sustain drug release. It was concluded that the optimal ratio of talc to be used is 1:4 (talc: Eudragit NE 30D). At this ratio, talc prevented any conglutination and enhanced the mechanical characteristics of the coating film to withstand the compaction forces applied during the tabletting process [56,57].

## 4. Microparticulate Systems for Compaction into Sustained Release Tablets (Microcapsules and Microspheres)

**Figure 3:**
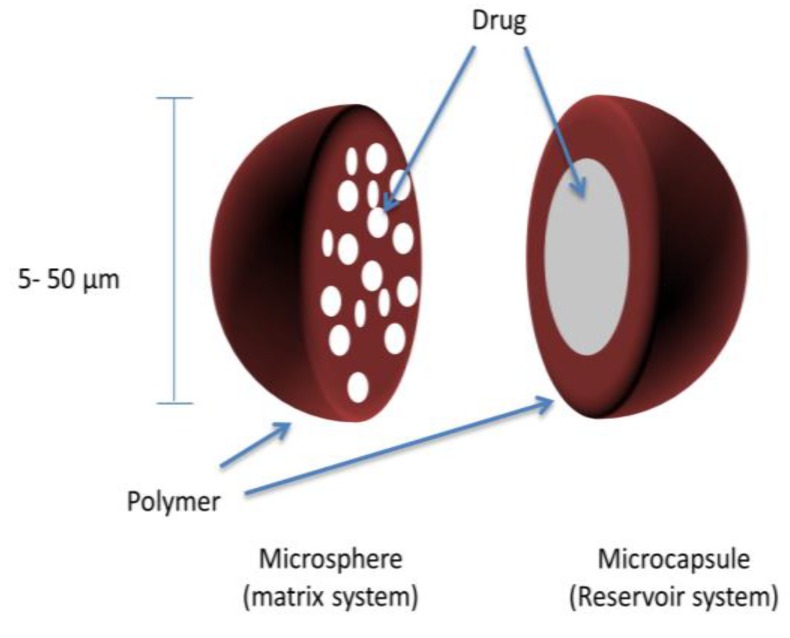
Microparticles (microspheres and microcapsules) matrix and reservoir systems.

Microcapsules are micromeritic reservoir systems, whereby the API is surrounded by a polymeric coating or shell (Figure 3). Similar to pellets, these microcapsules (coated multiparticulates) can then be compressed into a tablet to produce a sustained-release multiparticulate system. The major difference between microcapsules and pellets is size; microcapsules are much smaller particles with diameters typically between 100–150 μm [58]. It is important that microcapsules are not confused with microspheres, which comprise drug particles dispersed in a polymeric matrix (matrix system) (Figure 3).

### 4.1. Sustained-Release Tablets Based on Coated Microparticles (Microspheres and Microcapsules)

#### 4.1.1. Coating Layer

The key purpose for surrounding drug-containing microcapsules with a polymer coating is to embrace a time-controlled or sustained-release characteristic. However, similar to coated pellets, the polymer film coating must be able to withstand the compressive force applied during tabletting.

The polymer coating must be chemically compatible with the microcapsule core and must not react with it. It should also be non-hygroscopic, and can produce a cohesive film surrounding the microcapsule. Moreover, the polymer coat should have the right combination of mechanical strength, elasticity, and thickness to allow for it to remain intact after compaction [59].

Alginate is one of the polymers that have an influence on tablets’ mechanical properties [60]. The chemical structure and the ratio of glucuronic and mannuronic acid (of salt form alginate) are affect the compaction performance. A low ratio of guluronic acid is associated with more elastic properties than with a low mannuronic acid ratio. Sodium alginate was found to possess lower plastic properties than potassium alginate. Sodium alginate produced compressed tablets with low rigidity and high elasticity [61].

Alternatively, Sawicki et al. used hot tabletting to compress coated microcapsules consisting of tramadol hydrochloride (TH) into a sustained release dosage form. Four different polymeric coating systems were explored (two aqueous dispersions and two organic solutions). Aquacoat ECD and Eudragit^®^ RS/Eudragit^®^ RL mixtures were chosen as the aqueous dispersions, and an ethanol solution of EC and acetone/isopropanol solution of Eudragit^®^ RS/Eudragit^®^ RL were chosen as the organic solutions. The polymers were coated onto the drug-loaded microcapsules by means of the fluid bed coating. All of the polymer films could be sprayed onto the TH-containing microcapsules, except for the aqueous dispersion of Eudragit^®^ RS/Eudragit^®^ RL mixture. The coated microcapsules were compressed into tablets using the hot tabletting method, which uses a much lower compression force (1 kN) and a temperature of 56 °C in comparison to traditional tabletting. Granules consisting of melted Polyethylene glycol (PEG) 6000 were combined with the coated TH microcapsules during compression. The addition of PEG 6000 was vital to enable good flow properties, which would be otherwise be affected by the tackiness of the heated tablet formulation. In vitro drug release studies carried out compared the percent release of TH from tabletted-coated microcapsules prepared by traditional tabletting (10 kN/25 °C) with tabletted coated microcapsules prepared by hot tabletting (1 kN/56 °C) over an eight-hour period. Overall, the tabletted microcapsules prepared by hot tabletting sustained the release of TH better than the ones prepared by traditional tabletting. Also, the dissolution characteristics of the hot tabletted microcapsules were similar to that of the commercial brand: Tramadol Retard tablets [62].

#### 4.1.2. Thickness/Quantity of the Coating Layer

Besides the type of polymer membrane that is used to coat drug-containing microcapsules, the amount of polymer coating is also a very important parameter. The thickness of the polymer can affect the deformation characteristics of these coated microcapsules upon compaction into tablets. A thicker polymer coating improves the plastic-elastic properties of coated microcapsules when compressed; therefore, plastic and elastic deformation is observed when the amount of polymer is increased. Once the sustained release tablets consisting of coated microparticles disintegrate upon oral administration, the individually coated microparticles should slowly release the drug over a longer period. By increasing the polymer content, the thickness of the coating layer also increases; thus, it takes even longer for the drug to diffuse through the thick coating layer, and the drug release rate is further decreased. Garekani et al. prepared sustained-release theophylline microparticles coated with different amounts of polymer using the spray-drying technique. The spray dried microparticles were prepared with drug-to-polymer (D:P) ratios of 1:1, 1:2, and 1:3, and were then compacted into tablets. Aqueous copolymer dispersion, Eudragit RS 30 D, was incorporated as one of the coating polymers in amounts of 16.7 g, 33.4 g, and 50 g. Meanwhile, Eudragit RS PO was incorporated in amounts of 5 g, 10 g, and 15 g, and was dissolved in ethanol to produce an organic solution as the second coating polymer. Dissolution studies showed that drug release from tabletted spray-dried microparticles using organic solutions of Eudragit RS in ethanol was significantly affected by the amount of polymer coating used. After a period of 5 h, compacted spray-dried microparticles with a D:P ratio of 1:1 released ~25% of theophylline, microparticles with a D:P ratio of 1:2 released ~20% of theophylline, and microparticles with a D:P ratio of 1:3 released just over 10% of theophylline. It was evident that the compacted spray-dried microparticles with the higher proportion of polymer coating exhibited a slower release of drug and sustained the release of theophylline over the period investigated. In contrast, drug release from tabletted spray-dried microparticles using an aqueous dispersion of Eudragit RS was unaffected by the amount of polymer used. An increase in the amount of polymer, and thus the thickness, failed to decrease the drug release rate; however, there was still a sustained release of drug with the dissolution profile being similar for all three drug-to-polymer ratios (1:1, 1:2, and 1:3). It is believed that the aqueous polymer dispersion of Eudragit RS 30 D failed to act as a polymer coat to encapsulate the theophylline microcapsules, and subsequently formed a micromeritic matrix system instead of a reservoir system [63].

Nazar et al. investigated the use of Eudragit RL 100 and HPMC as potential coating polymers to retard the release of nimesulide. The polymer-coated nimesulide microcapsules were then compressed into tablets to produce a sustained release multiple-unit dosage form. The two polymers were used in combination in varying proportions (Eudragit:HPMC) to assess whether using different amounts of polymer caused more or less damage to the coated microcapsules during compaction, and subsequently, the effect that this had on the drug release rate. In vitro dissolution studies clearly showed that increased proportions of Eudragit in comparison to HPMC when used in combination sustained the release of the drug significantly over the 12-h period investigated [64].

Ahmad et al. prepared diclofenac sodium (DFS) microencapsulated tablets in which different ratios of EC were incorporated as the polymer coating. Three tablet formulations (F) were produced with drug-to-polymer (D:P) ratios of 1:1 (F1), 1:2 (F2), and 1:3 (F3). From their results, it was very clear that by increasing the ratio of EC from 1:1 to 1:3, there was a more sustained drug release pattern. An increase in the amount of EC gave rise to a thicker polymer coating that failed to rupture upon compaction into tablets [65]. The increased amount of EC effectively rendered the microcapsule impermeable to the dissolution medium, thus resulting in a slow release of the drug. A similar approach was adapted in the study of Ahmad et al., which used different ratios of Eudragit S 100 as a polymer coating for theophylline-loaded microcapsules. The drug-to-polymer (D:P) ratios used were 1:1 (F1), 1:1.5 (F2), and 1:2 (F3), and the microcapsules were then compressed into tablets. The in vitro drug release from the tabletted microcapsule formulations was compared with drug release from the commercially available sustained release Quibron-T/SR tablet. After a period of 5 h, drug release from F1 was approximately 70%; however, just over 40% of the drug was released from formulations F2 and F3 [66].

The mechanical properties of microcapsules were investigated in numerous studies. Kim et al. studied the characteristics of alginate-chitosan protein microcapsules. The Young’s modulus along with the releasing rate of the protein was measured for microcapsules coated with chitosan (1%, 2%, and 3%) using the microscale compression analysis. The results revealed that the microcapsule had values of 11.8 ± 4.9, 32.6 ± 11.4, and 42.8 ± 14.4 kPa of Young’s modulus when coated with chitosan at 1%, 2%, and 3% respectively. The increase in Young’s modulus was accompanied by a delaying of protein release [67].

#### 4.1.3. The Excipients

Fillers, which are also referred to as diluents, are the most the common excipients used in conjunction with polymer-coated microcapsules during compaction into sustained release tablets. The primary function of fillers is to essentially add bulk to low-dose tablets, so that the tablets that are manufactured are of a suitable and convenient size [47]. However, another key function of fillers when compressed with coated microcapsules is to act as a cushioning agent or protective agent to ensure that the functional polymer coat remains unruptured upon compression. Ideally, the cushioning agent should undergo plastic deformation upon compression, which as already discussed results in a permanent shape change, but not fragmentation. As well as deforming plastically, the cushioning agent should also deform much more readily than the coated microparticles. The filler should also produce sustained-release tablets of adequate mechanical strength that allow rapid disintegration upon oral administration, and most importantly, by providing protection to the polymer coat during compression, it must have no effect on the drug release kinetics [68].

Another study conducted by Lin et al. looked at the use of lactose as a potential cushioning agent to protect polymer-coated microparticles. Commercially available Surelease^®^ was used to coat chlorphenramine microparticles. Micronised lactose (ML) co-processed with hydroxy propyl cellulose (HPC), HPMC, and PVP by spray-drying was investigated for use as a novel cushioning agent. Previous research reported that the particle size of lactose was very significant in terms of its ability to act as an effective cushioning agent; a reduction in the particle size of lactose caused an increase in the cushioning effect. Therefore, very small particles of lactose (<10 µm), known as micronised lactose, were used in this study. The co-spray drying of micronised lactose with HPC proved to be the most effective at enhancing the cushioning effect, and increasing the concentration of HPC from 5% to 15% led to an even greater cushioning effect and this was reflected by an increase in the mean dissolution time (MDT) of the model drug. Co-spray drying ML with 5% HPMC caused a significant reduction in the cushioning effect, and this was reflected by an approximate 32% reduction in the MDT value. Meanwhile, co-spray drying ML with 5% PVP caused a slight reduction in the cushioning effect with an approximate 9% reduction in the MDT of chlorpheniramine [69].

Almaya and Aburub showed that the presence of lubricant (magnesium stearate 0.5%) has an effect on the compaction properties of MCC of grade Avicel PH-200 powder, where it has been noticed that compaction strength is less reliant on the particle size without adding a lubricant where the tensile strengths range between 4.59 MPa to 4.39 MPa for small particle size (<74 μm) and large particle size (180–250 μm) MCC powders, accordingly. However, compaction strength is highly affected by the particle size if the lubricant was used, where the tensile strength values range between 4.13 MPa and 2.55 MPa for small particles and large particles, respectively. While in the compaction of the dibasic calcium phosphate dihydrate, which is one of the fragile excipients, the compressibility strength demonstrated a slight change in the presence or absence of lubricant where the tensile strength values were 2.31 MPa and 1.93 MPa for small and large particles, respectively [70].

#### 4.1.4. The Compaction Force

Cantor et al. prepared coated microbeads consisting of cimetidine as the model drug. Surelease and Eudragit NE 30 D were the two aqueous polymeric dispersions used to coat the drug-loaded microbeads. These coated microbeads were then compressed into tablets to produce a sustained release multiparticulate system. Three different compression forces were investigated—200 kg, 400 kg, and 800 kg—to assess whether optimising the force used during the tabletting of coated microcapsules had any impact on the rupture characteristics of the polymer coating, and therefore the drug release profile. Tabletted Eudragit NE 30 D-coated microbeads exhibited a slow release of cimetidine over the 8-h period investigated at all of the compression forces when compared with tabletted Surelease-coated microbeads. Within 8 h, the drug release from compressed Eudragit NE 30 D-coated microbeads at compression forces of 200 kg, 400 kg, and 800 kg was approximately 15%, 15%, and 17% respectively. Within the same time period, the drug release from compressed Surelease-coated microbeads at compression forces of 200 kg, 400 kg, and 800 kg was approximately 22%, 26%, and 21%, respectively. These results show that Eudragit NE 30 D was more effective at sustaining the drug release compared with Surelease. This could be explained by the polymer coat experiencing minimal damage when compressed, and there were relatively small changes in the amount of drug released when the compression force was increased from 200 kg to 800 kg [71].

## 5. Sustained-Release Tablets of Coated Nanocapsules

**Figure 4:**
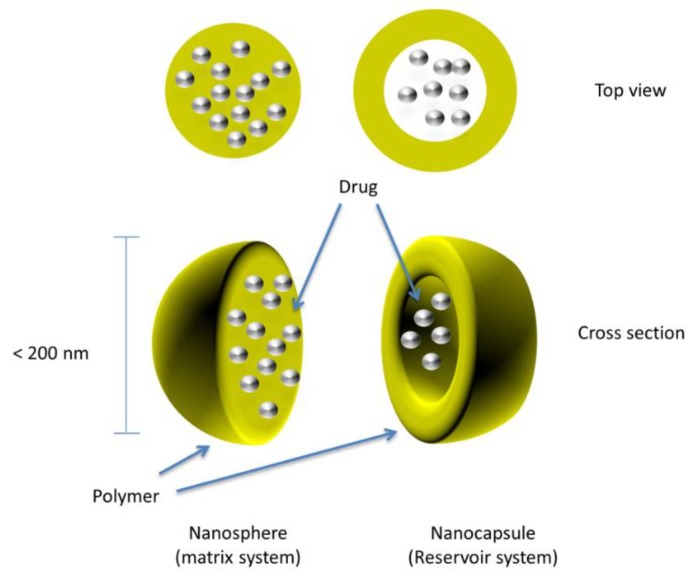
Drug-loaded coated nanospheres/nanocapsules.

The tabletting of nanoparticles and nanocapsules to form a sustained-release multiparticulate system is currently being explored. Nanocapsules are submicron (<1 µm) particles in which the drug-confined core is surrounded by a polymeric coat or shell, as shown in Figure 4.

Similar to pellets and microcapsules, there should be rapid disintegration of the tablet upon oral administration causing the release of individual drug-loaded nanocapsules, which should distribute readily along the gastrointestinal tract with a sustained release of the drug substance from each nanocapsule. Nanocapsules are fine particles and are much smaller compared to microcapsules and pellets; therefore, they tend to have greater bioavailability, better distribution in the gastrointestinal tract, and a reduced risk of local irritation and toxicity [72]. Friedrich et al. prepared drug-loaded polymeric nanocapsules containing dexamethasone, which is a corticosteroid used to reduce inflammation, as the model drug. These polymeric nanocapsules were then compressed into tablets to produce an oral sustained-release multiparticulate dosage form. Conventional dexamethasone tablets, without polymer-coated nanocapsules, were also prepared as a control to compare the in vitro drug release of both types of tablet [73]. Dissolution studies showed a much slower drug release from the nanoencapsulated tablets when compared to the conventional tablets, which suggested that the polymer coat of the nanocapsules remained intact and unruptured upon compaction, and this was further affirmed by scanning electron microscope (SEM) images, which showed the cross-section of the tablets in which intact polymeric nanocapsules were observed.

Chen YD et al. developed sustained-release orodispersible tablets (SR-ODTs) consisting of drug-loaded chitosan nanocapsules via direct compression in which prednisolone, which is also a corticosteroid, was incorporated as the model drug to treat asthma in paediatric patients. The concept behind SR-ODTs involves the rapid disintegration of the tablet in the oral cavity without the use of water to swallow the tablet, which therefore improves patient compliance, especially in the case of paediatric and geriatric patients [74]. The individual nanocapsules should then slowly release the drug over an extended period of time. In vitro dissolution studies indicated a fast drug release over a short period with over 80% of prednisolone released within 30 min from the tabletted chitosan nanocapsules, which indicated that there was damage to the chitosan-coated nanocapsules upon compression. However, when the amount of MCC 101, which was incorporated as a tabletting excipient, was increased from 20% to 40%, there was a decrease in the drug release rate. MCC 101 is a vital excipient as a filler/diluent within tablet formulations, but the direct compressibility property is what makes MCC 101 so important. By increasing the amount of MCC 101, the cushioning or protecting effect also increases, and thus this helps protect the integrity of the polymeric drug-loaded nanocapsules upon their compaction into tablets [75]. 

Similarly, Elwerfalli et al. also produced SR-ODTs comprising drug-loaded chitosan nanoparticles in which promethazine (PMZ) was incorporated as the model drug. Three different polymers were investigated as polymeric film coatings to help sustain the release of PMZ from the tabletted chitosan nanoparticles (CS-NPs). These were PEG, PVP, and polyethylacrylic acid (PEAA). Conventional ODTs containing PMZ, and not consisting of polymer-coated nanoparticles were used as a control as well as non-coated chitosan PMZ-loaded nanoparticles to compare the in vitro drug release with the polymer-coated CS nanoencapsulated SR-ODTs [72]. Dissolution studies revealed that ODTs containing polymer-coated chitosan nanoparticles could sustain the drug release, and therefore exhibited a much slower release of the drug over the 24-h period investigated compared to the conventional PMZ tablets and the uncoated chitosan nanoparticle tablets. The control tablets (i.e., conventional ODTs) failed to sustain the release of PMZ, with approximately 46% of the drug released within 20 min, and almost all of the drug released in 1 h (97.3%). ODTs comprising uncoated chitosan nanoparticles were able to sustain the drug release to some extent, with approximately 36% and 69% of drug released after 2 h and 6 h, respectively. Tablets containing PEG and PVP-coated nanoparticles exhibited a significantly slower drug release, with approximately 14% and 8% of PMZ released within 6 h, and less than half of the drug was released within the 24-h period investigated for both PEG-coated and PVP-coated NPs. Therefore, this indicated that the polymeric coating remained intact during compression, allowing for a slow release of PMZ over an extended period. However, this was not the case for tablets consisting of PEAA-coated nanoparticles, in which 45% of drug was released within 2 h, causing a burst effect [72].

In another study conducted by Usman et al., polymer-coated nanoparticles containing lornoxicam as the model drug were prepared using four different polymers: HPMC, Na-alginate, pectin, and PVP. These coated drug-loaded nanoparticles were subsequently compressed into tablets, and the in vitro drug release was assessed and compared with the dissolution rate of conventional lornoxicam tablets. It was concluded that the tabletted-coated nanoparticles could sustain the drug release more effectively as well as exhibiting an improved oral bioavailability than the conventional tablets [76].

Elzoghby et al. attempted to produce sustained-release floating tablets comprising alfuzosin hydrochloride (ALF)-loaded casein nanoparticles via direct compression. The drug-loaded casein nanoparticles were prepared using the spray-drying technique; prior to this, the ALF-casein was mixed with genipin. The spray-dried ALF-casein nanoparticles were then directly compressed into tablets. Three different tablet formulations were prepared (T1, T2, and T3), each with differing concentrations of genipin: 0%, 10%, and 40% *w/w*. In vitro tablet dissolution studies demonstrated a sustained release of ALF over the 24-h period investigated. The drug release after 24 h for tablet formulations T1, T2, and T3 were 99.77%, 93.57%, and 78.44% and the mean dissolution time (MDT) of ALF was 6.94 (T1), 7.76 (T2), and 8.25 (T3) h, respectively. Therefore, it could be concluded that tablets with a higher concentration of genipin were able to prolong the release of the model drug more effectively. Tablet formulation T3, with a genipin concentration of 40% *w*/*w*, released around 21% less of the drug within 24 h, and had a MDT that was approximately 1.3 h greater than that of tablet formulation T1, which had no genipin present at all [77].

## 6. Evaluation of the Compaction Behaviour of Particulate Systems

The compaction of pharmaceutical powder and particles takes place in three main stages, including filling the die with powder, compressing of the powder, and obtaining the compacted powder. Initially, during powder compression, the applied pressure is low, and this enables the particles to come close to each other and compress, leading to a reduction in powder volume and porosity. This occurs until the particle reaches a maximum structure that can be attained without further movement; when the applied pressure elevates, a further reduction in powder volume takes place along with the change in the particles’ size [78]. In order to evaluate the powder mechanical properties, it is significantly important to examine the powder behaviour in each stage. Different theories and analysis methods were used to characterize the powder mechanical strength, including: Leuenberger model, Heckle model, Shapiro model, and Panelli-Filho model. The widely used models are summarised as in Table 2.

## 7. Conclusions

Oral modified-release multiparticulate dosage forms are becoming a more effective therapeutic alternative to conventional immediate-release dosage forms, as they provide a slower, sustained drug release and minimise local irritation in the gastrointestinal tract. The former eliminates the need for multiple daily-dosing regimens, thus significantly improving patient compliance. Nonetheless, the tabletting of coated multiparticulates remains a challenge, as the compaction force exerted on coated pellets, microparticles, or nanoparticles often results in damage to the polymeric coating, and subsequently the rapid release of the drug substance. Therefore, it is important that various formulation and process variables are optimised to ensure that the coating polymer can withstand compression and remain intact during the tabletting process. The most important variable is the type of polymer that is chosen for the coating of drug-loaded particulates, which must have the right combination of plasticity and elasticity to remain unruptured upon compression. Additional research is required in this field, but current published work reveals very promising results, and the compression of drug-loaded nanocapsules into sustained-release tablets has the potential to become one of the most popular oral multiple-unit modified release dosage forms.

## Figures and Tables

**Figure 5:**
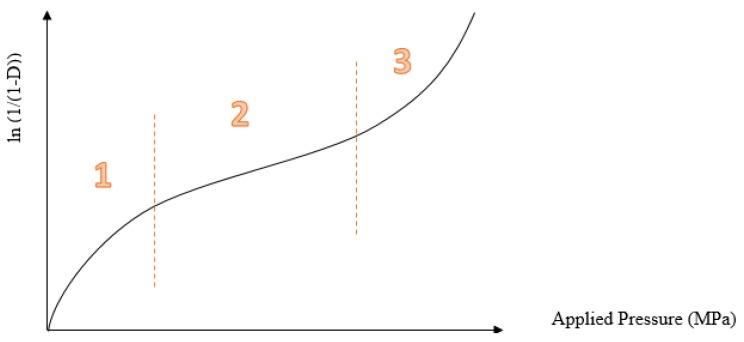
Heckel profile represents the relationship between porosity and applied pressure: the first part (1) is particle rearrangements and fragmentation, the second part (2) is the elastic and plastic deformation, and the third part (3) is the effect of elastic deformation.

**Figure 6:**
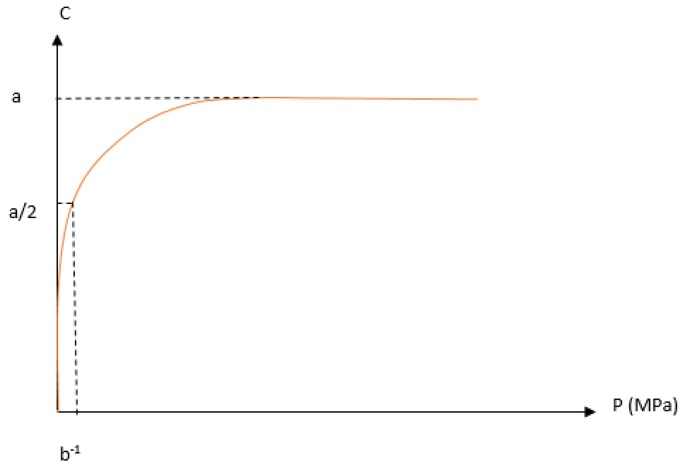
The illustration of the strain pressure curve, in which C represents volume reduction, and P is the applied pressure; a and b are constants according to Kawakita model.

**Table 1:** Pharmaceutical polymers used in the coating of particulate systems with their elongation value and chemical structures.

Polymer Lass	Coating Polymer	Chemical Structure	Elongation Value	References
Cellulosic Polymers	Ethyl cellulose	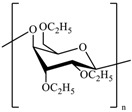	<5%	[20]
	Microcrystalline cellulose	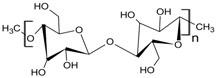	10.8%	[21]
	Hydroxypropyl methylcellulose	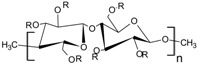 R= H, CH_3_, CH_2_CH(CH_3_)OH	≈40%	[20,21]
Acrylic polymers	Eudragit NE 30 D	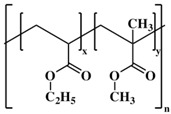	≥365%	[22]
	Eudragit RS 30 D	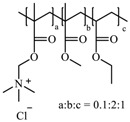	<50%	[4]
	Eudragit RL 30 D	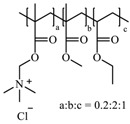	<50%	[4]
Polyvinyl acetate	Kollicoat^®^ SR 30 D	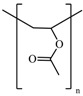	1.1%	[23]

**Table 2:** Methods of characterization of pharmaceutical powder before and during the compression process: direct and indirect methods.

	Method of Evaluation	Type of Method	Characterization	Reference(s)
DIRECT METHOD	Microscopic Evaluation	Scanning Electron Microscopy	Morphology and surface characteristic of particles and pellets	[79]
	Particle Size Analysis	Sieve Analysis	Size of the particles and pellets of pharmaceutical compounds	[80]
INDIRECT METHOD	Analytical Procedures	Dissolution Studies (In Vitro)	Release profile(s) of active pharmaceutical ingredients	[81]
	Texture Analysis	Texture Analyser	♦ Elastic–plastic properties of powders and pellets. ♦ Disintegration of compacts of fast-dissolving formulation ♦ Mucoadhesion properties of polymers	[82]
	Compression Analysis	Heckle Model	♦ Powder compressibility–porosity relationship ♦ Elastic–plastic deformation (Figure 5)	[83]
	Compression Analysis	Kawakita Equation	♦ Flowability and cohesiveness of bulk powder ♦ Volume–compressibility relationship (Figure 6) ♦ Powder rearrangement at low pressure	[84]
	Compression Analysis	Partial Differential Equations	♦ Prediction and evaluation of the variables influencing mechanical properties ♦ Determine shape model of pharmaceutical tablets	[85]
	Numerical Compression Analysis	Finite Element Method	♦ Evaluation of tablets shape and their mechanical properties	[86]
